# Intelligent Optimization of Tower Crane Location and Layout Based on Firefly Algorithm

**DOI:** 10.1155/2022/6810649

**Published:** 2022-06-29

**Authors:** Cong Liu, Fangqing Zhang, Xiaojian Han, Hongyu Ye, Zanxi Shi, Jie Zhang, Tiankuo Wang, Jianjun She, Tianyue Zhang

**Affiliations:** ^1^College of Civil Engineering, Nanjing Tech University, Nanjing 211816, China; ^2^Urban Planning and Design Institute, China Design Group Co., Ltd., Nanjing 210014, China; ^3^School of Architecture, Southeast University, Nanjing 210096, China

## Abstract

The existing tower crane positioning layout mainly depends on the experience of construction personnel, and the best tower crane positioning can be found through a large number of manual data calculation. This manual method is time-consuming and impractical. In view of this, aiming at the current situation that building information modeling (BIM) software can only obtain the relative coordinates of components, this article puts forward the key technology of importing computer-aided design (CAD) graphics into geographic information system (GIS) software to automatically obtain the world coordinate information. By clarifying the transfer relationship between the component material supply point, the component initial positioning point, and the tower crane optional positioning point, as well as the cooperative relationship between each positioning point and the tower crane operation, the tower crane positioning optimization model is formed, and the firefly algorithm is used to automatically calculate and generate the best positioning layout method of the tower crane on the project site. In this study, the vertical transportation and positioning of components are studied, and intelligent construction is formed by integrating information technology. It can further enrich the functions of perception, analysis, decision-making, and optimization; realize the decision-making intelligence of industrial buildings; and achieve the organic unity of engineering construction execution system and decision-making command system.

## 1. Introduction

Tower crane is an essential equipment for the vertical transportation, installation, and positioning of prefabricated components in high-rise residential buildings. Because there are many types of components transported by tower crane, such as prefabricated components, reinforcement, formwork, and scaffold, and the location of material supply is complex, the positioning and layout planning of tower crane in the construction site of high-rise residence is a common construction technical problem.

The previous research on the optimal positioning method of tower crane mainly adopts two methods. The first method is to minimize the cross area between tower cranes. Zhang et al. proposed a computer model to optimize the position of a group of tower cranes to determine the minimum crossing area [1]. Irizarry and Ebrahim integrated geographic information system (GIS) and building information modeling (BIM) to determine the location of the minimum crossing area of tower crane [2].

The second method is to minimize the lifting time and cost of the tower crane. Tam et al. and Wilson K. W. Chan proposed the optimization of supply points around tower cranes based on a genetic algorithm [3]. Huang et al. proposed the optimization of tower crane and material supply location in high-rise building site based on mixed-integer linear programming [4]. Lien and Cheng used particle swarm optimization algorithm to determine the location of tower crane considering the optimization of material supply and demand [5]. Sohn et al. developed a stability-based optimal selection and supporting design management method of tower crane and proposed a method to optimize economic feasibility (cost) on the premise of meeting lifting conditions and stability [6]. Orozco et al. corrected the linearization technique [7]. Balcazar et al. compared the proportional integral differential regulator, the first-order sliding mode regulator, and the second-order sliding mode regulator to adjust two different types of mathematical models [8]. In order to find the optimal travel cost of all crane operations in a given time period, Marzouk and Abubakr proposed tower crane type selection decision support based on building information modeling and genetic algorithm [9]. Moussavi Nadoushani et al. proposed an improved crane location optimization model, which not only minimizes the total cost of lifting operation, but also considers the impact of crane location on its required capacity and operation time [10]. Vazirinia and Kaveh compared the effects of enhanced vibrating particle system (EVPs) in solving the actual tower crane layout problem through the performance of particle swarm optimization (PSO) and four newly developed metaheuristic algorithms, collision volume optimization (CBO), enhanced collision body optimization (ECBO), and vibrating particle system (VPS). The results show that ECBO is superior to the other three methods in both cases [11]. Younes and Marzouk tested the impact of conflict between tower cranes, the maximum efficiency of tower crane layout, and quantitatively evaluated the impact of tower crane operation conflict on the total operation time and cost through agent-based simulation (ABS) model [12].

Sebt et al. described the application of GIS technology [13] in construction site planning, including the acquisition of spatial data mainly used in the location optimization of tower crane, and determining the feasible task area according to the location of demand point and supply point and the working radius of crane. The geometry of the construction site is generated by CAD (computer-aided design) tools, which will determine the appropriate combination of tower cranes to optimize the location. The output of the GIS model includes one or more feasible areas covering all demand and supply points, which are then linked to the GIS tool and generate an integrated layer to visualize the optimal location of the tower crane. Wang et al. developed an integrated method combining building information modeling (BIM) and firefly algorithm (FA) [14] to automatically generate the optimal layout plan of tower crane. Zhu et al. also proposed an open source method (OSA) [15], which retrieves the geometric information in IFC through the spatial structure of IFC (i.e., IFC tree) and converts it into shape file by developing automatic polyhedron generation algorithm (AMG). OSA can connect BIM and GIS more stably and efficiently by strengthening the data conversion from BIM to GIS. Dasović et al. used BIM model to input local coordinates from BIM into excel table and converted local position coordinates into global coordinates according to Croatia map projection reference coordinate system HTRS96/TM [16].

At present, some metaheuristic algorithms play an important role in solving complex construction problems. Metaheuristic algorithm mainly includes the monarch butterfly optimization (MBO) [17], slime mould algorithm (SMA) [18], moth search algorithm (MSA) [19], hunger games search (HGS) [20], Runge Kutta method (RUN) [21], colony predation algorithm (CPA) [22], and Harris hawks optimization (HHO) [23]. These algorithms simulate various phenomena and processes in nature and human thinking activities to guide the whole search process.

Through the study of the above literature, it is found that the positioning layout of tower crane has gradually changed from manual calculation to intelligent calculation. Most of the research related to tower crane machinery depends on the application of mathematical programming formulas. These methods aim to minimize the cost and time of transferring components. However, the main problems of these studies are: (1) there is a lack of research on the method of obtaining coordinates. Some studies use the relative coordinate system, which is contrary to the universal use of the world coordinate system in the construction process. Some articles simply do not provide coordinate data, which raises questions about the authenticity of the calculation. Since the coordinates of component material supply point, component positioning point, and optional tower crane positioning point are closely related to the mathematical model of tower crane operation, the steps and methods of obtaining world coordinates should be studied first. (2) The research on the existing intelligent optimization algorithm has been in-depth enough, and articles used to optimize the positioning and layout of tower crane are also common. (3) At present, the output results after the optimization of tower crane positioning and layout mostly adopt the expression of plane graphics, which is the lack of three-dimensional visual expression.

In view of this, this article presents a method of using CAD and GIS integration to obtain coordinate information and establish tower crane positioning and layout system. The specific contributions are as follows:

1. This article puts forward the key technology of importing CAD graphics into GIS software to automatically obtain the world coordinate information.
2. By clarifying the transfer relationship among the component material supply point, the component initial positioning point, and the tower crane optional positioning point, as well as the cooperative relationship between each positioning point and the tower crane operation, the tower crane positioning optimization model is formed.
3. The optimal positioning and layout method of tower crane in the project site is automatically generated by firefly algorithm.

The rest of this article is organized as follows: In Section 2, the method of transforming CAD and GIS into world coordinates are introduced. In Section 3, we provided a case study describing the mathematical model of tower crane operation and the operation steps of firefly algorithm. In Section 4, we show the calculation results of the case in Section 3 and analyze and visually simulate them. Finally, some conclusions are drawn in Section 5.

## 2. Methodology

### 2.1. Conversion of CAD and GIS into World Coordinates

Geographic information system (GIS) is defined as a system for storing, retrieving, and analyzing geographic reference data. It is a tool for analyzing geographic environment, mainly two-dimensional [24]. CAD is a system for drawing and updating maps. It is a tool for analyzing the internal environment of individual buildings, including two-dimensional and three-dimensional [25]. The coordinate system is a reference system for measuring horizontal and vertical distances on a map [26]. The coordinate system used by GIS is mainly the geographic coordinate system based on longitude and latitude, which is used to evaluate the location of real-world features. The coordinate system used by CAD is usually based on Cartesian coordinate system [27]. The difference between the coordinate systems used in GIS and CAD is that the geographic coordinate system uses longitude and latitude to represent the horizontal and vertical position relative to the center of the earth, while the Cartesian coordinate system uses a set of simpler axes to locate the position relative to any origin. Often, CAD coordinates need to be converted into GIS coordinates [28].

The CAD software developed by Autodesk company can directly obtain the relative coordinates of the project site. The LocaSpace viewer software [29] developed by Zhongketuxin company can have a good data transfer interface for the DWG format file output by CAD, avoiding complicated data conversion operations. The world coordinates of components can be queried and obtained through the following simple steps. The conversion of material supply points is taken as an example: (1) The CAD general drawing is simplified and unnecessary information is deleted. (2) After selecting the base point of the simplified general drawing and setting the export unit, the wblock command is called to write the whole general drawing into blocks and exported the world coordinate point table. (3) LocaSpace viewer4 is opened. (4) Clicked on toolbox ⟶ data conversion ⟶ batch conversion of plane coordinates to longitude and latitude. (5) The processed CAD world coordinate excel table is added, the relevant parameters are set, and started the conversion. (6) The longitude and latitude coordinates of the converted material supply point are obtained.

### 2.2. Integration of CAD and GIS

As today's organizations become more complex and global, there is often a need to promote better decision-making through a variety of graphics and database applications [30]. CAD software allows organizations to view the details of a single drawing, while GIS allows organizations to view multiple sites with less detail [31]. As a decision support system, GIS can explain and solve practical problems in various fields such as land resource management, surveying and mapping, urban planning, market analysis, geology, and hydrology [32]. CAD applications usually focus on design, which is suitable for the scale of buildings or infrastructure engineering, and usually lack the reliable attribute model. Both software have specific purposes, but with the increasing demand for comprehensive planning, design and management of natural resources, and infrastructure assets, it is usually necessary to visualize or analyze internal and external data at the same time, so as to propose CAD/GIS integration, which is realized by developing a seamless interface, which integrates different views into a single world and a single perspective [33]. ESRI (Environmental Systems Research Institute) is currently the largest GIS software developer. Their ArcGIS application ArcMap reads CAD drawings and directly uses DXF files, which is similar to adding GIS layers to CAD systems [34].

Since the DWG file compatible with lsv4 needs to have a coordinate system, the DWG file exported from CAD is projected by using ArcGIS software [35], and the projection and longitude and latitude coordinates are imported into lsv4. The specific steps are as follows: (1) open ArcGIS software, connect to the target folder, add DWG file ⟶ export to SHP format and load layer ⟶ set the required projection coordinate system ⟶ convert to CAD using arctoolbox conversion tool. (2) Select import CAD in lsv4, import the DWG file in Step (1), and add two control points according to the principle of geographic registration. (3) Load CAD into GIS to realize the integration of CAD and GIS.

Through this method, the optimal location of tower crane and the actual location of material supply point at the construction site of a residential project will be determined conveniently, quickly, and accurately. The high-rise residential project is located in Hai'an city. The construction section is composed of four 29-storey residential buildings, covering an area of about 12000 square meters and a total building height of 99 meters.

**Figure 1:**
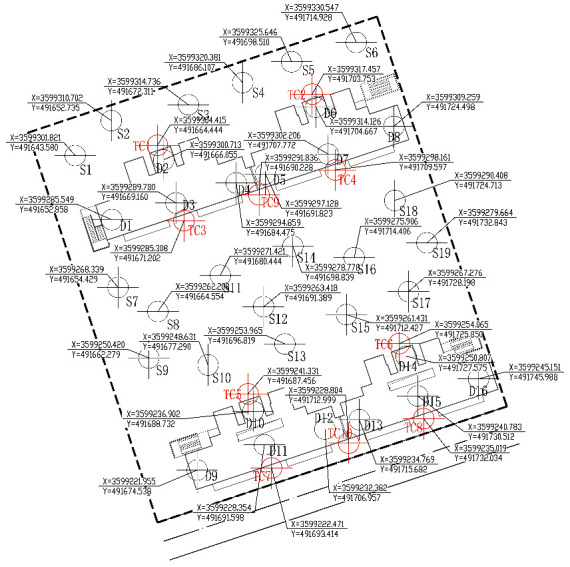
Optional material supply point, tower crane coordinates, and site coordinates of initial positioning point of fixed components of Hai'an project.

Around the site around the four main residential buildings, a total of 19 material supply points can be provided for stacking and storing prefabricated components, reinforcement, formwork, and scaffolding. These 19 points are marked as S1, S2,…, and S19, respectively, as shown in Figure 1. At the same time, each floor of the residential project can provide 16 preliminary positioning points of components (D1, D2,…, and D16) (Figure 1).

After the tower crane transports the components to one of the initial positions, it will unload immediately for the next lifting. The tower crane is only responsible for the initial positioning of components, and the accurate positioning and installation of components is carried out by the on-site construction personnel with auxiliary equipment with more convenient operation and more accurate positioning. The advantage of this operation is to improve the lifting efficiency of the tower crane and shorten the time consumed in the accurate positioning of components. Even for 19 material supply points and 16 initial positioning points of components, there are hundreds of millions of possible combinations due to the need for four tower cranes to operate one-to-one in the middle, and the optimal solution cannot be easily obtained. For attached tower cranes, they are usually placed near fixed structures that do not hinder other construction activities. Therefore, the four tower cranes have 10 alternative positions (TC1, TC2,…, and TC10). The positioning layout of tower crane obtained by intelligent optimization algorithm will be marked as T1, T2, T3, and T4.  Table 1 lists the coordinates of 19 optional material supply points (s) in GIS.  Table 2 lists the coordinates of 16 fixed initial positioning points (d) of components in GIS.  Table 3 lists 10 optional tower crane positioning layout (TC) coordinates in GIS.

### 2.3. Solution

Firefly algorithm (FA) was first studied by Xin She Yang of the Engineering Department of the University of Cambridge in 2009 based on the behavior of fireflies. Firefly algorithm is a heuristic algorithm inspired by the flickering behavior of fireflies. The algorithm has many advantages, such as easy to understand, few parameters, and easy to implement, and has been applied in many fields. It has great potential in solving nondeterministic polynomial problems (NP) [36].

## 3. Case Study

### 3.1. Technical Route

**Figure 2:**
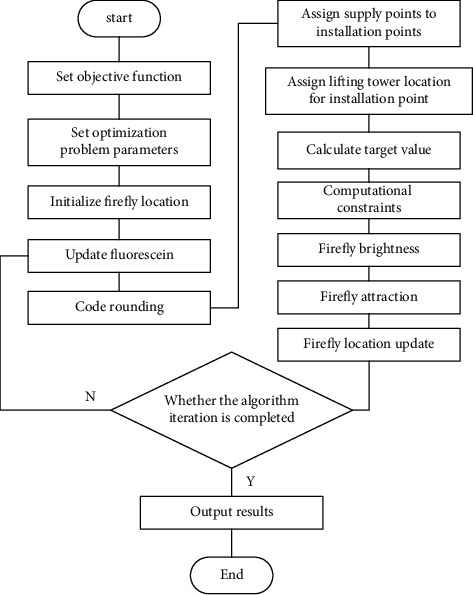
Flow chart of the proposed algorithm.

The road map is shown in Figure 2. First, the firefly algorithm sets the objective function; sets the parameters of the problem to be optimized according to the objective function; initializes the position of the firefly; and sets the number of firefly populations, iteration times, initial fluorescein concentration, and maximum and minimum attraction and absorption coefficient. Then, the position of fireflies in the space defined by the objective function is initialized, their fluorescein is updated, and a new generation of firefly population is obtained. Then, the optional supply point (s), the optional tower crane positioning point (TC), and the fixed component initial positioning point (d) are coded by double-layer coding. The first layer codes the optional supply point (s) for the component initial positioning point (d), and the second layer codes the tower crane positioning point (TC) for the component initial positioning point (d), calculates the value of the objective function, and judges whether the constraints of the objective function are met. The calculation for firefly brightness is firefly brightness = (1/objective function), which is equivalent to fitness. The absorption coefficient will be multiplied by brightness to get firefly attraction after sorting. The firefly's position update formula needs to be calculated and moved accordingly before selecting the qualified firefly and judging whether the iteration has been completed. If the algorithm has completed the global optimization, the cycle is ended and the results are outputted, otherwise return to updating the fluorescein for optimization calculation.

### 3.2. Operation Steps

Fireflies can determine the existence and attraction of other individuals by sensing the intensity and frequency of luminescence of other fireflies within the effective range, so as to attract the opposite sex and prey.

The firefly algorithm is based on the following three rules:

1. All fireflies are gender neutral, which means they are attracted to other fireflies not because of male or female.
2. The attraction of fireflies is directly proportional to their brightness. Therefore, for any two flickering fireflies, the firefly with weaker brightness will move towards the firefly with stronger brightness. The higher the brightness means that the smaller the distance between the two fireflies, the greater the distance, the lower the brightness, and the smaller the attraction. If there is no firefly brighter than the current firefly, it will move randomly.
3. The brightness of the firefly is determined by the value of the objective function (fitness). For the maximization problem, the brightness can be directly proportional to the value of the objective function. For the minimization problem, the brightness is inversely proportional to the value of the objective function.

First, *n* randomly distributed fireflies in the space are solved. Each firefly has its own perceptual search radius. The brightest firefly is searched within the search radius and moved towards it, then the search radius and attraction are updated, and continued to search and move until the number of iterations is reached. The firefly will gather around the brightest firefly or on the brightest firefly points it can perceive. The position of these points is the optimal solution of the objective function. After designing the objective function, the specific operation steps of firefly algorithm are as follows:

**Step 1 .**

Initialize the location of the firefly

Set the number of firefly population, number of iterations, initial fluorescein concentration, and maximum and minimum attraction and light absorption coefficient.

**Step 2 .**

Update fluorescein and generate population

**Step 3 .**

Update the location of fireflies

Update the position and quantity of fireflies, set the attraction coefficient, and the smaller the objective function, the more attractive the firefly will be. The larger the objective function, the more attractive the firefly will be. Limit the range of independent variables.

**Step 4 .**

Calculate the maximum brightness and attraction of fireflies

Calculate firefly brightness = (1/objective function), which is equivalent to fitness. According to the brightness ranking of fireflies, brightness = 1/objective function. After ranking, the absorption coefficient will be × Brightness, get firefly attraction.

**Step 5 .**

Select and judge whether the iteration of the algorithm is completed

According to the “for Gen = 1: maxge… End” loop statement in Step 4, select the firefly with large objective function value and judge whether it meets the constraints. If so, end the loop, otherwise return to Step 2.

**Step 6 .**

Output results

According to the algorithm results, the iterative curve of firefly algorithm and the layout of tower crane are drawn. The iterative curve continues to decline, indicating that the algorithm is effective, and the algorithm converges only when the curve finally flattens.

### 3.3. Condition Preset

In order to establish the mathematical model of tower crane positioning layout, some preset conditions are listed below:

1. All alternative material supply points, tower crane positioning and layout points, as well as the model of tower crane are preset.
2. For the supply point *s* and the initial positioning point D of each group of tower cranes, the transportation capacity is known. For example, the total number of tower cranes, the number of components to be lifted on each floor, the maximum load and minimum load of tower crane, the maximum working radius of tower crane jib, and the minimum working radius of jib. The demand of the initial positioning point of each component is also preset.
3. The construction duration of the work area is roughly the same.
4. The material transportation from each group's supply point *s* to the initial positioning point D of the component is completed by only one tower crane.
5. When each tower crane transports components between supply point *s* and component positioning point D, it must be within the allowable weight radius circle of the tower crane, which is jointly determined by the jib length and lifting capacity of the tower crane. More than one tower crane is required for the construction of the project. Since the coordinates of demand points are relatively fixed, more research will focus on the possible positioning and layout of supply points and tower cranes.

### 3.4. Problem Solving

#### 3.4.1. Establish Mathematical Model of Tower Crane Operation

In this study, the purpose of establishing the mathematical model is to calculate the position of material supply point and tower crane from the preset positioning point according to the minimum lifting time and cost. The objective function is shown in formula (1) [14]. In order to maximize the effectiveness of the tower crane, it is necessary to understand the operating mechanism of the tower crane, master the following parameters, and make overall analysis.

(1) $$\begin{array}{c} \min TC=\min\sum_{k=1}^{K}\sum_{i=1}^{I}\sum_{j=1}^{J}T_{ij}^{k}\times Q_{ij}^{k}\times C^{k}, \end{array}$$

where *K* is the number of tower cranes, *I* is the number of material supply points, and *J* is the number of initial positioning points of components. *T*_*ij*_^*k*^ is the delivery time of the *k* tower crane from the *i*th supply point (*S*_*i*_) to the *j*th component initial positioning point (*D*_*j*_), which is calculated by equations (4)–(11). *Q*_*ij*_^*k*^ is the lifting capacity of the *k* tower crane from the *i*th supply point (*S*_*i*_) to the *j*th component initial positioning point (*D*_*j*_). *C*^*k*^ is the unit time cost of operating the *k*th crane.

Assuming that *Q*_*S*_*i*__ is the total supply of the *i*th supply point (*S*_*i*_), according to the relationship that the lifting capacity of all tower cranes is less than the total supply, there is the following conditional constraint formula:

(2) $$\begin{array}{c} \sum_{K=1}^{K}\sum_{j\mathrm{=1}}^{I}Q_{ij}^{k}\leq Q_{S_{i}}. \end{array}$$

Assuming that *Q*_*D*_*j*__ is the total demand of the *j*th component positioning point (*D*_*j*_), according to the fact that the lifting capacity of all tower cranes is equal to the total demand of each supply point, there is the following conditional constraint formula [14]:

(3) $$\begin{array}{c} \sum_{k=1}^{K}\sum_{i=1}^{I}Q_{ij}^{k}=Q_{D_{j}}. \end{array}$$

**Figure 3:**
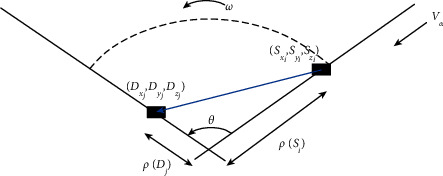
Horizontal movement mechanism of tower crane hook.

**Figure 4:**
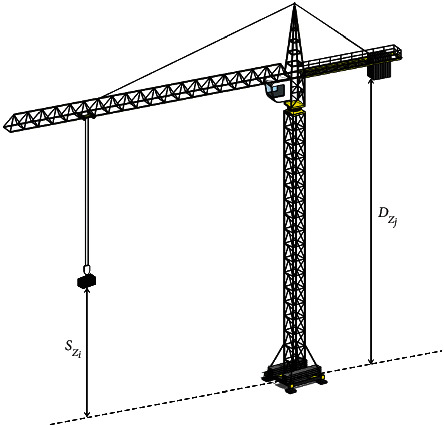
Vertical movement mechanism of tower crane hook.

Figures 3 and 4 show the free movement of the hook of the tower crane in the horizontal and vertical directions, which determine the total time and cost of an installation task. (*S*_*x*_*i*__, *S*_*y*_*i*__, *S*_*z*_*i*__) and (*D*_*x*_*i*__, *D*_*y*_*i*__, *D*_*z*_*i*__) refer to the coordinates of the supply point and the initial positioning point of the component for a single lifting task, respectively. (*C*_*x*_*k*__, *C*_*y*_*k*__, *C*_*z*_*k*__) represents the positioning layout coordinates of the *k*th tower crane. The mathematical model of tower crane operation is shown in the following formulas [4]:

(4) $$\begin{array}{c} T_{h}^{k}\mathrm{=max}\left(T_{\alpha }^{k},T_{\omega }^{k}\right)+\alpha \mathrm{\times min}\left(T_{\alpha }^{k},T_{\omega }^{k}\right), \end{array}$$

(5) $$\begin{array}{c} T_{ij}^{k}\mathrm{=max}\left(T_{h}^{k},T_{v}^{k}\right)+\beta \mathrm{\times min}\left(T_{h}^{k},T_{v}^{k}\right), \end{array}$$

(6) $$\begin{array}{c} T_{v}^{k}=\frac{\left|D_{zj}-S_{zi}\right|}{V_{h}^{k}}, \end{array}$$

(7) $$\begin{array}{c} T_{\alpha }^{k}=\frac{\left|\rho \left(S_{i}\right)-\rho \left(D_{i}\right)\right|}{V_{\alpha }^{k}}, \end{array}$$

(8) $$\begin{array}{c} T_{\alpha }^{k}=\frac{1}{V_{\omega }^{k}}\mathrm{\times arccos}\left(\frac{l_{ij}^{2}-\rho {\left(D_{j}\right)}^{2}-\rho {\left(S_{i}\right)}^{2}}{2\times \rho \left(D_{j}\right)\rho \left(S_{i}\right)}\right). \end{array}$$

For any lifting, the transportation distance of the hook can be calculated by the following formulas [4]:

(9) $$\begin{array}{c} \rho \left(D_{i}\right)=\sqrt{{\left(D_{x_{j}}-C_{x_{k}}\right)}^{2}+{\left(D_{y_{i}}-C_{y_{k}}\right)}^{2}}, \end{array}$$

(10) $$\begin{array}{c} \rho \left(S_{i}\right)=\sqrt{{\left(S_{x_{j}}-C_{x_{k}}\right)}^{2}+{\left(S_{y_{i}}-C_{y_{k}}\right)}^{2}}, \end{array}$$

(11) $$\begin{array}{c} 1_{ij}=\sqrt{{\left(D_{x_{j}}-S_{x_{i}}\right)}^{2}+{\left(D_{y_{i}}-S_{y_{i}}\right)}^{2}}, \end{array}$$

where *T*_*h*_^*k*^ is the horizontal transportation time of the hook of the tower crane at position *k*, which is composed of *T*_*α*_^*k*^ and *T*_*ω*_^*k*^. *T*_*α*_^*k*^ is the movement time of the hook in the radial direction (along the radius of the circle), and *T*_*ω*_^*k*^ is the movement time of the hook in the tangential direction (along the tangent direction of the circle). *T*_*v*_^*k*^ is the vertical movement time of the hook at *k* position. *α* and *β* are two parameters between 0 and 1. *α* indicates the matching ability of the hook to move in the radial and tangential directions in the horizontal plane, and *β* reflects those matching abilities in the vertical and horizontal directions. When *α*=0, the radial and tangential directions of the hook move synchronously *α*=1, the radial and tangential step-by-step movement of the hook depends on the skill level of the operator and the tolerance of the site. When *β*=0, the hook moves synchronously in the vertical and horizontal directions *β*=1. The hook moves step by step in the vertical and horizontal directions, which also depends on the skill level of the operator and the tolerance of the site.

#### 3.4.2. Setting Firefly Algorithm Parameters

The firefly algorithm simulates the information transmission behavior of fireflies in the space defined by the objective function by idealizing some luminous characteristics of fireflies. It has the following four important parameters to be set:

*(1) Luminous Intensity (LI)*. *LI* is the luminous intensity of fireflies, which decreases with the increase of the distance between them. *LI* is determined by the target value in its position. The brighter the firefly, the better its position and the greater its attraction. Generally, the luminous intensity is related to the objective function. Since this simulation is to calculate the minimum value of the objective function, we define *LI* as the reciprocal of the objective function. See the following formula [14] for details.

(12) $$\begin{array}{c} LI_{i}=\frac{1}{f\left(x_{i}\right)}=\frac{1}{\min TC},\\ x_{i}=\left(x_{i,1},x_{i,2},L,x_{i,k},x_{i,k+1},L,x_{i,d}\right),\;i=1,2,L,n. \end{array}$$

Each *x*_*i*_ is a feasible vector, that is a feasible tower crane positioning and layout solution. In this vector, the first *k* objects (*x*_*i*,1_, *x*_*i*,2_, *L*) represent *k* alternative tower crane positions, and the remaining objects (*x*_*i*,*k*+1_, *L*, *x*_*i*,*d*_) represent (d-k) alternative material supply point positions. Each object has a value of “0” or “1.” “1” means to select, “0” means not to select.

*(2) Attraction between Fireflies*. In the firefly algorithm, the attraction function *β* is a monotonically decreasing function, such as the following generalized calculation formula [37]:

(13) $$\begin{array}{c} \beta =\beta_{0}e^{-\gamma^{r_{ij}^{n}}},n\geq 1, \end{array}$$

where *r* is the distance between two fireflies. *β*_0_ is the attraction when *r* = 0. When *r* reaches a certain level, fireflies will no longer be attractive to each other, so *β* ≥ *β*_min_. *γ* is the light absorption coefficient, *γ*=1.

*(3) The Distance between Fireflies*. The Cartesian distance between any two fireflies *i* and *j* at *x*_*i*_ and *x*_*j*_ is shown in the following formula [37]:

(14) $$\begin{array}{c} r_{ij}=\left\|x_{i}-x_{j}\right\|=\sqrt{\sum_{k=1}^{d}{\left(x_{i,k},x_{j,k}\right)}^{2}}. \end{array}$$

*(4) The Movement of Fireflies*. Entering the stage of firefly movement, firefly *i* will be attracted by another more attractive (brighter) firefly *j* and move towards it. The movement of firefly is determined by the following formula [37]:

(15) $$\begin{array}{c} x_{i}^{\prime }=x_{i}+\beta \times \left(x_{j}-x_{i}\right)+\alpha \left(\text{rand}-\frac{1}{2}\right). \end{array}$$

#### 3.4.3. Setting the Operation Parameters of Tower Crane

**Figure 5:**
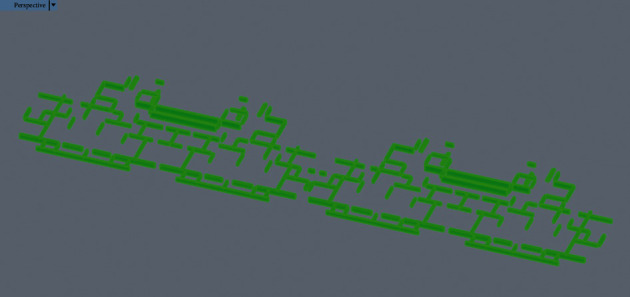
Beam components to be lifted on each floor of Hai'an residence.

Because the construction site of high-rise buildings has different layout methods in different construction stages, this study mainly focuses on the tower crane layout of four residential buildings in the same construction section. In the process of prefabricated construction, the material with the largest transportation volume of tower crane is precast reinforced concrete components. According to the component quantity information provided by the BIM model, it is easy to obtain the number of times that the tower crane of a standard construction section (including 4 residential buildings) needs to be lifted for the construction of the first floor. For this project, 128 components need to be initially positioned on each floor of a residential building, a total of 4. Therefore, the total statistical result is that 512 precast concrete components need to be lifted on each floor, as shown in Figure 5.

According to the specifications and construction experience of tower cranes, each tower crane can be lifted and lowered 4 times per hour, and each tower crane can work for 8 hours. In addition, the construction completion time of a standard layer is 5 days. Therefore, the number of tower cranes required for the project is: cell [512 ÷ (4 × eight × 5)] = 4. The “cell” function is used to round up the calculation results to the nearest integer multiple for the following reasons: (1) the number of tower cranes must be an integer; (2) the less the number of tower cranes, the lower the cost; and (3) the lifting capacity provided by the tower crane must be greater than the lifting demand. Considering the site restrictions, boom length, and rated lifting capacity of the tower crane, four TC6026 tower cranes are selected.  Table 4 shows the main performance parameters of this type of tower crane.

## 4. Results and Analysis

Relevant parameters in the firefly algorithm are set as required: initial fluorescein concentration *α*=0.5, initial attraction *β*_0_=1, minimum attraction *β*_min_=0.2, light absorption coefficient *γ*=1, the number of firefly population is 100, and the maximum number of iterations is 100. According to the established objective function formula (1), the luminous intensity calculation formula (12) is obtained, and the firefly algorithm code is written with MATLAB r2021b update5 software for optimization calculation. In order to verify the method, the modeling process was checked with the project leader to ensure that there was no omission of data and no error in input. The calculation results of the mathematical model were discussed and finally provided to the project construction party as a preferred scheme.

**Figure 6:**
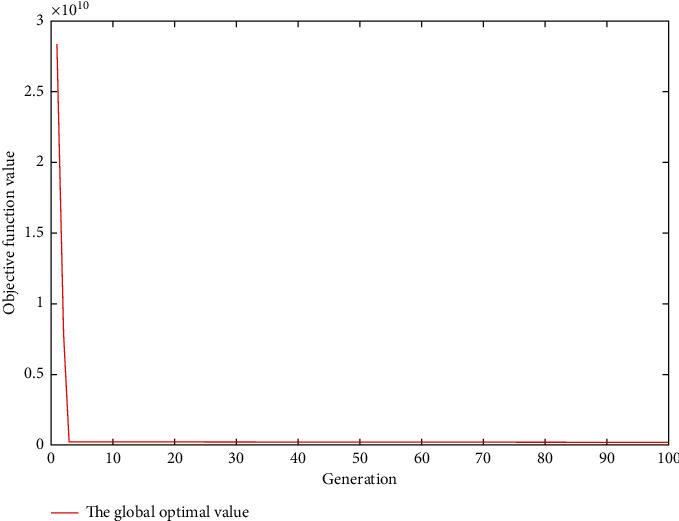
Iterative curve of firefly algorithm optimization.

**Figure 7:**
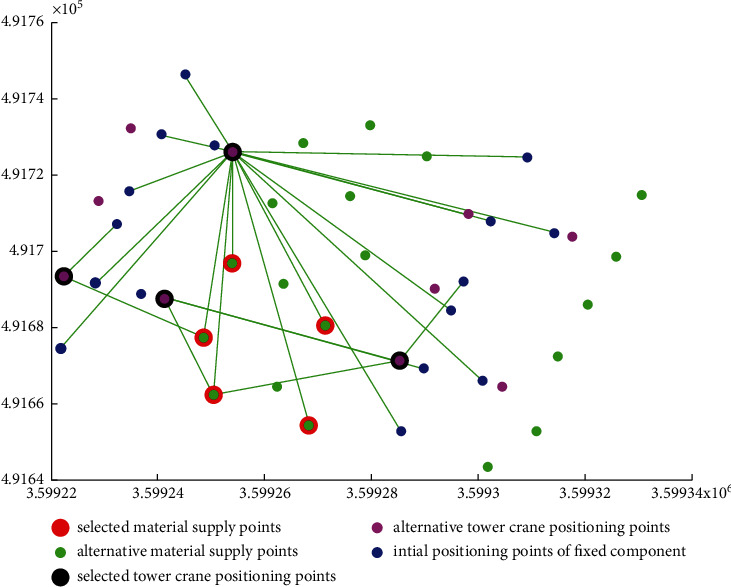
Layout of fireflies.

**Figure 8:**
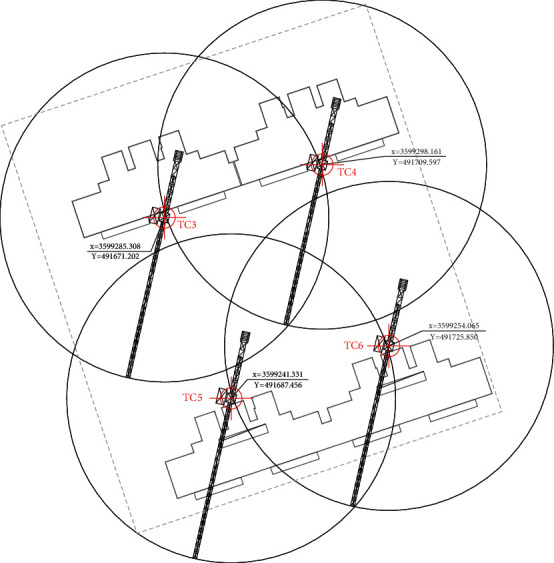
Final positioning points of tower crane.

Table 5 shows the coordinates of the five material supply points finally selected, Table 6 shows the coordinates of the four tower crane positioning points finally selected, and Figures 6 and 7 show the performance of the algorithm in this case. The algorithm selects four positioning layout points TC3, TC4, TC5, and TC6 to install the tower crane. The global optimal value is obtained around the fifth generation. The objective function mintc = 236918118.675023, and the push is 0. The positioning layout of the final positioning layout points of the four tower cranes in the GIS software is shown in Figure 8, and the total lifting time is 25355613.747098 s.

The positioning and layout of tower crane on the construction site of high-rise residence is a common construction technical problem. According to the construction environment and construction conditions of Hai'an high-rise residence site, in view of the problem that only relative coordinates can be obtained in CAD software and the world coordinate information of positioning points cannot be obtained directly, the combination of project CAD model and GIS topographic map is adopted to obtain 19 component material supply points quickly and accurately, world coordinates of 16 component initial positioning points, and 10 optional tower crane positioning points.

According to the characteristics of tower crane operation, the coordinate information is associated with the mathematical model of tower crane operation to form the objective function. Facing the needs of multipoint selection, the evaluation index is established, and the positioning and layout points of four tower cranes on the project construction site are finally determined by using the firefly algorithm. Through the visual output of the calculation results in the GIS software, the prefabricated components can be lifted in place quickly and safely within the construction period, saving the operation cost.

## 5. Conclusions

Because many tower cranes are affected by the above uncertain material supply points and component positioning points, the positioning layout of tower cranes is often considered to be a complex combination problem. The existing tower crane positioning layout mainly depends on the experience of construction personnel, and the best tower crane positioning can be found through a large number of manual data calculation. This manual method has large input, is time-consuming, and not practical. In order to solve these problems, this article proposes a method of using the integration of CAD and GIS to obtain coordinate information and establish the positioning and layout system of tower crane. This system can make full use of various component information in the whole life cycle of BIM model, including spatial data, positioning coordinates, construction period data, cost data, and maintenance data. Then, the intelligent optimization algorithm is used to combine GIS and intelligent optimization algorithm. Using the component material supply point, component positioning point, and optional tower crane positioning point obtained by GIS, a multi-objective optimization model is established to automatically calculate and generate the best positioning layout of tower crane on the project site, so as to improve the positioning and assembly efficiency of components. In this article, GIS and CAD topographic maps are combined to obtain the world coordinates of components, and BIM software is used for simulation. This method may also be combined with GIS and BIM. With the deepening of follow-up research, it is believed that the data transmission standards between GIS and BIM software will be more accurate and perfect, which will help give full play to the advantages of their respective platforms. In the future work, we compare with other algorithms to further verify the superiority of firefly algorithm.

## Figures and Tables

**Table 1:** Coordinates of alternative material supply point (s).

	Coordinate (X)	Coordinate (Y)	Coordinate (Z)
S1	3599301.821	491643.580	0
S2	3599310.702	491652.735	0
S3	3599314.736	491672.311	0
S4	3599320.381	491686.107	0
S5	3599325.646	491698.510	0
S6	3599330.547	491714.928	0
S7	3599268.339	491654.429	0
S8	3599262.208	491664.554	0
S9	3599250.420	491662.279	0
S10	3599248.631	491677.290	0
S11	3599271.421	491680.444	0
S12	3599263.418	491691.389	0
S13	3599253.965	491696.819	0
S14	3599278.778	491698.839	0
S15	3599261.431	491712.427	0
S16	3599275.906	491714.406	0
S17	3599267.276	491728.198	0
S18	3599290.408	491724.713	0
S19	3599279.664	491732.843	0

**Table 2:** Coordinates of initial positioning points of fixed component.

	Coordinate (X)	Coordinate (Y)	Coordinate (Z)
D1	3599285.549	491652.858	80000
D2	3599300.713	491666.055	80000
D3	3599289.780	491669.160	80000
D4	3599294.859	491684.475	80000
D5	3599297.128	491691.823	80000
D6	3599314.126	491704.667	80000
D7	3599302.206	491707.772	80000
D8	3599309.259	491724.498	80000
D9	3599221.955	491674.538	80000
D10	3599236.902	491688.732	80000
D11	3599228.354	491691.598	80000
D12	3599232.382	491706.957	80000
D13	3599234.769	491715.682	80000
D14	3599250.807	491727.575	80000
D15	3599240.783	491730.512	80000
D16	3599245.151	491745.988	80000

**Table 3:** Alternative tower crane positioning layout (TC) coordinates.

	Coordinate (X)	Coordinate (Y)	Coordinate (Z)
TC1	3599304.415	491664.444	0
TC2	3599317.457	491703.753	0
TC3	3599285.308	491671.202	0
TC4	3599298.161	491709.597	0
TC5	3599241.331	491687.456	0
TC6	3599254.065	491725.850	0
TC7	3599222.471	491693.414	0
TC8	3599235.019	491732.034	0
TC9	3599291.836	491690.228	0
TC10	3599228.804	491712.999	0

**Table 4:** Main performance parameters of TC6026 tower crane.

Parameter	Value
Maximum working radius of boom	60 m
Minimum working radius of boom	2.5 m
Maximum load	8.0 t
Minimum load	2.6 t

**Table 5:** Coordinates of selected material supply points.

	Coordinate (X)	Coordinate (Y)	Coordinate (Z)
S7	3599268.339	491654.429	0
S9	3599250.420	491662.279	0
S10	3599248.631	491677.290	0
S11	3599271.421	491680.444	0
S13	3599253.965	491696.819	0

**Table 6:** Coordinates of selected tower crane positioning points.

Tower crane no.	*X*	*Y*	*Z*	Select results	Lifting time (s)
T1	3599285.308	491671.202	0	TC3	1386624.8438171
T2	3599298.161	491709.597	0	TC4	1782787.85242233
T3	3599241.331	491687.456	0	TC5	5152019.24629754
T4	3599254.065	491725.850	0	TC6	17034181.8045609
Total					25355613.747098

## Data Availability

The data used to support the findings of this study are available from the corresponding author upon request.
